# Uses of Bovine Pericardium in General Surgery

**DOI:** 10.1111/ans.70833

**Published:** 2026-07-06

**Authors:** William A. Ziaziaris, Makaela Bowman, Paul D. Morris, Kilian G. M. Brown, Michael J. Solomon, Jerome M. Laurence

**Affiliations:** ^1^ Department of Colorectal Surgery Royal Prince Alfred Hospital Sydney NSW Australia; ^2^ Institute of Academic Surgery (IAS), Royal Prince Alfred Hospital Sydney NSW Australia; ^3^ Surgical Outcomes Research Centre (SOuRCe), Royal Prince Alfred Hospital Sydney NSW Australia; ^4^ Department of Upper Gastrointestinal Surgery Royal Prince Alfred Hospital Sydney NSW Australia; ^5^ Faculty of Medicine and Health, Central Clinical School, The University of Sydney Sydney NSW Australia

**Keywords:** bovine pericardium, clinical application, general surgery, tissue properties

## Abstract

**Background:**

Bovine pericardium (BP) is increasingly utilised in complex surgical reconstruction because of its favourable biological and mechanical properties. Although widely used in cardiovascular surgery, its role in general surgery remains less well characterised.

**Methods:**

A narrative review was conducted to synthesise available evidence regarding the biological properties, preparation methods, tissue integration and clinical applications of BP in general surgery.

**Results:**

BP demonstrates favourable tensile strength, low antigenicity, and reliable biocompatibility. Available evidence, largely from observational studies and small case series, supports its use across a variety of general and subspecialty surgical settings including vena cava and portal vein reconstruction, diaphragmatic hernia repair, abdominal wall reconstruction, and arteriovenous fistula formation. Compared with synthetic grafts, BP may offer advantages in contaminated or infected fields, although comparative data is limited. Tissue integration varies according to preparation technique, with decellularised grafts demonstrating improved cellular infiltration and reduced calcification. Novel applications, including reconstructing the empty pelvis after exenteration, are emerging.

**Conclusion:**

BP is a durable and individually adaptable biomaterial with expanding applications in general surgery. Its favourable handling characteristics and biological integration make it a valuable adjunct in complex reconstructions, although further studies are required to better define its long‐term outcomes and comparative effectiveness.

## Graphical abstract

Bovine pericardium is a versatile biologic material with expanding applications across general surgery.

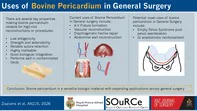

## 1 Introduction

BP has been widely used in cardiac and vascular surgery for decades; however, its application in general surgery remains comparatively under‐recognised [1, 2, 3, 4]. With increasing complexity of surgical reconstruction and a growing need for biologic materials that perform reliably in contaminated or high‐risk environments, BP may offer distinct advantages [1, 2, 3, 4]. Despite this, the literature describing its use across general surgery remains fragmented, so this review aims to summarise the biological properties, preparation techniques, and current and emerging uses of BP in general surgery.

## 2 Properties and Preparation of Bovine Pericardium

### 2.1 Processing and Preparation

The harvest, cleaning and chemical processing of BP grafts vary. In the majority of products, after harvest from cattle, fat and connective tissue are manually removed from raw pericardium and the remaining tissue is cleaned. Grafts are then rinsed with purified water, where blood and soluble proteins are removed prior to chemical processing [5, 6]. Oxidative bleaching destroys non‐collagen proteins while preserving the collagen structure of the BP graft [6].

Crosslinking of BP grafts forms covalent bonds between collagen molecules, increasing tissue strength and stability, limiting biodegradation and reducing antigenicity [3]. Traditionally this has been achieved with aldehyde crosslinking, most commonly glutaraldehyde; however, free aldehyde may cause cytotoxicity and inflammation [7, 8]. Processes that neutralise or remove aldehydes may produce grafts with comparable mechanical and biochemical properties with better endothelialisation and compatibility with host tissue [7, 9]. Dye mediated photo‐oxidation is an alternative to aldehyde crosslinking, stabilising collagen in bovine pericardium without toxic aldehyde byproducts [10]. Some preliminary data suggests that photo‐oxidation may lead to greater mechanical strength and biocompatibility with reduced calcification compared with aldehyde crosslinked grafts [10, 11].

### 2.2 Mechanical and Biological Properties

The final product is an acellular graft devoid of animal proteins and DNA, ensuring reduced antigenicity with similar extensibility to human connective tissue [3, 12]. Additionally, processing of BP allows for consistent graft thickness of 0.5 mm, allowing for optimal surgical handling, reproducible reconstructions and suture retention [13]. Various commercially available products, their preparation and properties, are outlined in Table 1.

**TABLE 1:** Summary of commercially available bovine pericardium grafts.

Company	Trade name	Preparation	Surgical preparation
Artivion _TM_	PhotoFix Decellularized Patch	Proprietary decellularisation; photo‐oxidation (aldehyde‐free)	Standard 0.9% saline rinse; keep moist; single use
Baxter	Supple Peri‐Guard	Sterilised, decellularised, glutaraldehyde‐crosslinked	Immerse in saline ≥ 3 min; keep moist; single use
Baxter	Veritas Collagen Matrix	Decellularised, non‐crosslinked	Immerse in saline up to 60 min; keep moist; single use
Baxter	Vascu‐Guard	Decellularised, glutaraldehyde‐crosslinked	Standard saline rinse; single use
Edwards	Bovine Pericardial Patch	XenoLogiX decellularised, glutaraldehyde‐crosslinked	Standard saline rinse; single use
Edwards	Duravess Vascular Patch	Decellularised with XenoLogiX fixation	Standard saline rinse; single use
FOC Medical	Surgifoc Pericardial Patch	Decellularised; Tutoplast‐style processing	Standard saline rinse; single use
LeMaitre	XenoSure Plus	Decellularised, glutaraldehyde‐crosslinked	Standard saline rinse; single use
LeMaitre	VascuCel Bioscaffold	Decellularised, glutaraldehyde‐crosslinked	Standard saline rinse; single use
LeMaitre/Admedus	CardioCel	ADAPT decellularisation; ultra‐low glutaraldehyde; anti‐calcification	Standard saline rinse; single use
Tutogen/B. Braun	Tutomesh	Decellularised; Tutoplast method	Standard saline rinse; single use

## 3 Biological Integration

The histological integration of BP grafts following implantation depends on the method of preparation. Glutaraldehyde‐fixed BP integrates primarily through fibrous encapsulation and formation of a thin fibro‐endothelial neointima at the host interface [14]. Aldehyde crosslinking renders the matrix impermeable to cells, limiting vascular ingrowth and provoking a chronic, low‐grade inflammatory response dominated by macrophages and giant cells [7, 15]. Over time, progressive calcification along aldehyde‐modified collagen leads to stiff, acellular tissue prone to late degeneration [15]. By contrast, decellularised BP supports true biological integration into host tissue with cell infiltration, neovascularisation and extracellular‐matrix remodelling [16, 17]. Fibroblasts and macrophages migrate into the scaffold, depositing new collagen and glycosaminoglycans, while capillaries extend into the graft [18, 19]. Over several weeks, a fibrovascular network replaces the acellular matrix, forming organised collagen bundles and an endothelialised surface with minimal calcification [17, 18, 19, 20].

## 4 Surgical Applications

### 4.1 Vascular Reconstruction

The inferior vena cava (IVC) may need partial or complete reconstruction during transplant or oncological procedures. Previous reports, including from our centre, have published good short‐term outcomes from both patch and tubularised bovine pericardium grafts for various indications [21, 22]. Portal vein (PV) reconstruction is commonly performed in the context of pancreaticoduodenectomy or total pancreatectomy and is occasionally required in liver transplantation. In cases of tumour involvement, PV resection may improve rate of R0 resection [23]. While partial resection may only require primary closure or patch repair, more extensive resections require either venous reconstruction with primary anastomosis or interposition grafts [24]. Options include autologous, cadaveric, biological or synthetic grafts. An international multicentre study demonstrated reduced portal vein thrombosis following interposition graft with BP versus synthetic grafts, though both autologous and cadaveric grafts performed better [25]. This study however also demonstrated significant heterogeneity of post‐operative anticoagulation between centres and venous reconstruction techniques [25].

While synthetic grafts such as polytetrafluoroethylene (PTFE) have increased risk of infection and require lifelong anticoagulation to maintain patency, BP offers long‐term patency and superior flexibility and adaptability allowing for individualisation [26]. BP is highly malleable and can be used for both patch and tubularised IVC and PV reconstruction (Figure 1). This can be performed over syringes, anal sizers, or other cylindrical products using vascular staplers, sized to the individual patient's IVC or PV. Additionally, patients with BP grafts at our centre routinely receive long‐term lower‐dose anticoagulation than typically required for PTFE to prevent thrombosis [22].

**FIGURE 1:**
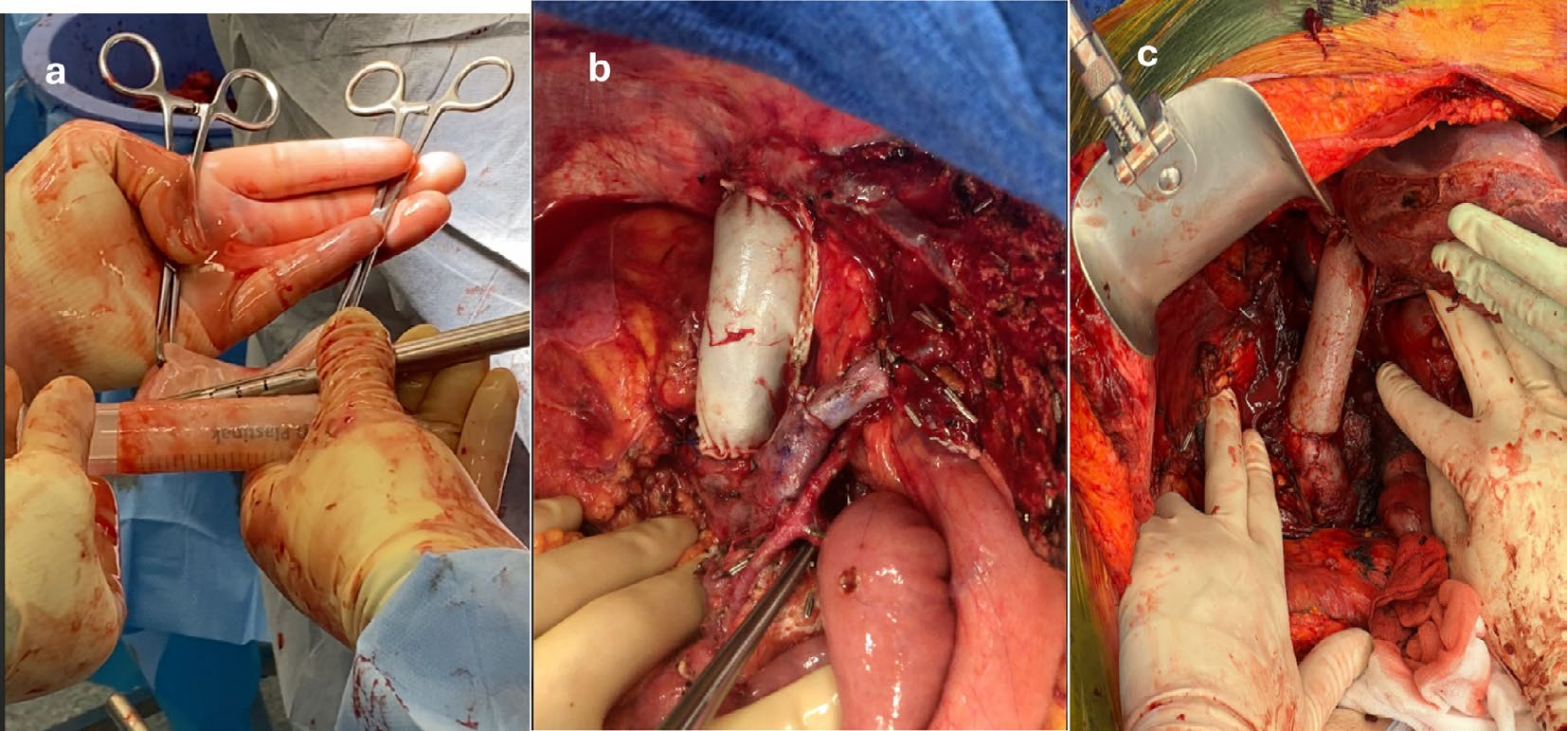
(a) Formation of tubularised bovine pericardium graft over a 30 mL cylindrical syringe using Lexington Aeon Vascular stapler (60 mm white reload) and Allis forceps. (b) and (c) Tubularised IVC graft inset with 4–0 Prolene after extended right hemihepatectomy for hilar cholangiocarcinoma and right nephrectomy with IVC involvement and en‐bloc resection respectively.

### 4.2 Diaphragmatic Hernia Repair

Diaphragmatic hernias pose a significant challenge to surgeons, with no clear guidelines or consensus for a superior method or prosthetic for repair post injury, hernia or oncological resection. Generally, acute traumatic hernias identified early are most likely amenable to primary repair via open or minimally invasive techniques. Larger hernias or defects following oncologic resection, however, may require interposition. A recent, large systematic review of 4595 patients demonstrated patch repairs of the diaphragm are common, used in up to one third of patients with 19 different patches used, of which Gore‐tex (PTFE) was the most common [27]. PTFE‐based products, however, lack elasticity and can shrink over time, causing significant scarring, though the degree to which this affects diaphragmatic function is unknown [28].

Use of BP for the management of iatrogenic, traumatic, and congenital diaphragmatic defects has been described previously [27, 29]. The World Society of Emergency Surgery (WSES) recommends biologic meshes in acutely incarcerated or strangulated hernias where there may be an increased risk of prosthetic infection or contamination [30]. Some series have reported improved outcomes with biologic compared with synthetic meshes, though case numbers remain low [31]. Moreover, BP grafts may be beneficial for these repairs as the elasticity and mobility of BP closely mimics the native diaphragmatic muscle and central tendon tissue. Unlike other biologic meshes, BP is pliable, malleable, and easily inset into diaphragmatic defects (see Figure 2).

**FIGURE 2:**
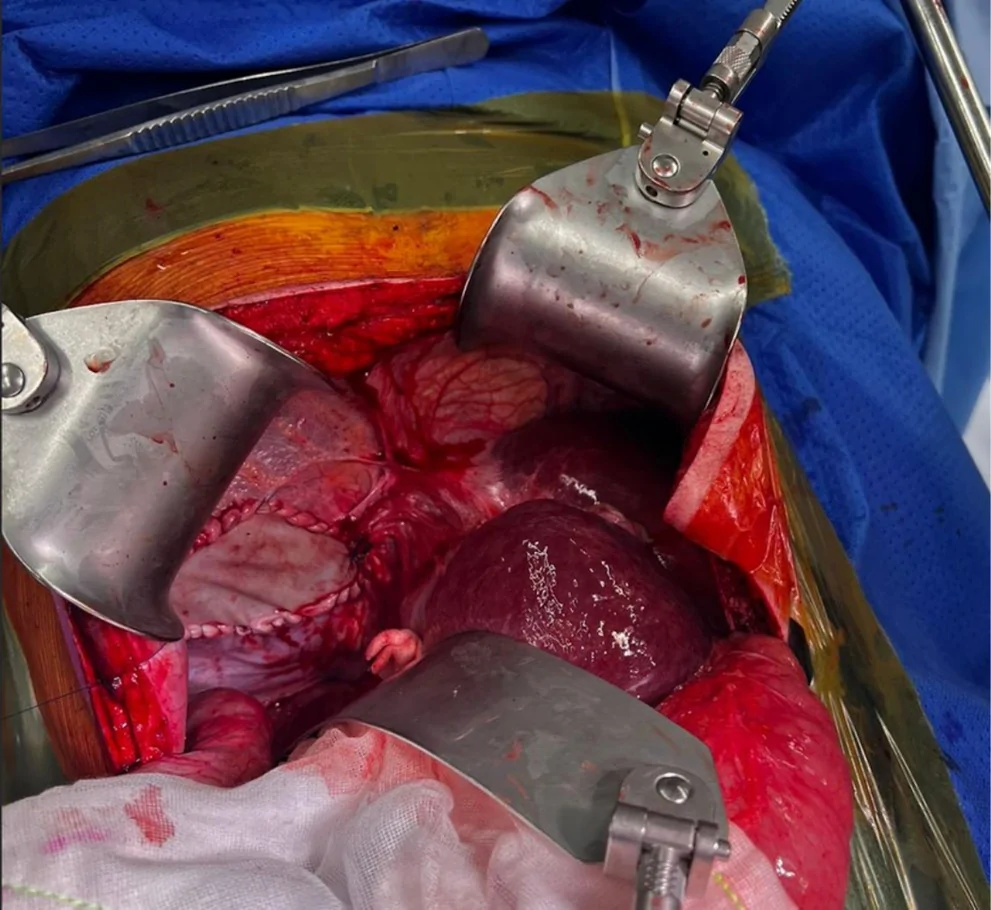
Bovine pericardium patch for the repair of a traumatic diaphragmatic hernia with delayed presentation containing both liver and colon.

### 4.3 Complex Abdominal Wall Reconstruction

Abdominal wall reconstruction for complex hernias requires a nuanced approach and planning individualised to the patient and their hernial anatomy. BP can be incorporated into hernia repair as follows:

– A biologic mesh either as a sublay, inlay, or onlay in elective, emergent, and/or contaminated fields
– Bridging mesh to facilitate retrorectus repair

In an early study assessing 2 preparations of bovine pericardium, glutaraldehyde‐treated grafts developed strong fibrous tissue replacement with good incorporation of the graft into the abdominal wall, whilst the lyophilised grafts were substantially resorbed within 12 weeks, with the resultant thin layer of fibrous tissue inadequate for abdominal wall support [32]. Experimental models comparing bovine pericardium to polypropylene mesh demonstrated reduced inflammatory reaction and better collagen fibre formation with BP, suggesting potential benefits for tissue integration over synthetic meshes [33]. Moreover, compared with other biologics (both human‐ and porcine‐derived), BP has a similar or better tissue adaptation profile, but studies directly comparing biologic grafts are rare [2, 33, 34]. A comparison of bovine pericardium with commonly used prosthetic and biologic materials in abdominal wall reconstruction is summarised in Table 2.

**TABLE 2:** Comparison of Bovine Pericardium with common prosthetic and biologic materials in abdominal wall reconstruction.

Material	Type	Mechanical strength	Infection tolerance	Resorption profile	Handling characteristics	Recurrence data	Cost considerations
Bovine Pericardium (BP)	Xenograft (biologic)	High tensile strength; can be tailored (patch or tubularised)	May be better tolerated in contaminated fields compared with synthetic mesh [1, 2, 33]	Non‐resorbable or slowly remodelling depending on processing	Pliable, easily shaped, good suture retention	Limited data; mostly small series and observational studies	High (especially for large sheets) [33]
Polypropylene Mesh	Synthetic (permanent)	High	Poor in contaminated fields; high infection risk if exposed	Non‐resorbable	Easy to handle; widely available	Strong long‐term data; low recurrence in clean cases	Low
PTFE	Synthetic (permanent)	Moderate–high	Poor; infection often requires explantation	Non‐resorbable	Less elastic; can be stiff	Acceptable recurrence; higher complication rates in contamination	Moderate–high
BioA	Synthetic (absorbable)	Moderate (temporary support)	Better tolerated than permanent synthetics in contamination	Fully resorbable (months)	Easy to use; scaffold‐like	Variable recurrence; higher than permanent mesh in some series [35]	Moderate
Phasix	Synthetic (biosynthetic, resorbable)	High initial strength with gradual loss	Designed for improved performance in contaminated fields; emerging evidence	Resorbed over 12–18 months	Good handling; similar to permanent mesh	Growing evidence; promising mid‐term outcomes	High
Porcine Dermis/ADM	Xenograft (biologic)	Moderate; variable depending on processing	Better tolerated in contaminated fields compared with synthetics	Gradual remodelling	Good handling; thicker than BP	Mixed results; higher recurrence in bridging repairs [36, 37]	High

*Note:* Cost categories are relative and may vary by institution and geographic location. Evidence is predominantly derived from observational studies and heterogeneous cohorts. Cost categories are relative estimates only. Published unit cost data for biological and biosynthetic materials are institution‐ and country‐specific, are not uniformly reported in the literature, and may vary substantially depending on sheet size required. For complex abdominal wall reconstruction requiring large sheets (up to 30 cm), BP in particular carries a high unit cost; however, formal health economic analyses comparing BP with alternative materials in this context are currently lacking and represent an important area for future study.

Abbreviations: ADM, acellular dermal matrix; PTFE, polytetrafluoroethylene.

#### 4.3.1 Use in Contaminated Fields

Another advantage of BP is its versatility and safety in contaminated fields. Animal studies and limited clinical data suggest that biologic grafts may demonstrate improved tolerance to infection compared with synthetic meshes; however, high quality, randomised evidence directly comparing these materials remains limited [1, 33, 38]. In their rat‐based infected hernia repair study, Harth et al. demonstrated improved but variable clearance of controlled *Staphylococcus aureus* contamination amongst biological grafts used for hernia repair compared with a synthetic polyester mesh control (Parietex) [1]. Similar results have been seen in rabbit‐based studies comparing acellular dermal matrix grafts to PTFE mesh [38]. While these findings are promising, current evidence is largely derived from experimental models and small clinical series and does not support routine superiority of BP over other biologic or synthetic materials in contaminated fields [1, 33, 38]. Further comparative studies are required.

#### 4.3.2 Retrorectus Repair

For ventral defects, retrorectus repair is increasing in popularity, providing a biomechanically superior repair with reduced recurrence and morbidity compared with onlay techniques [39]. Complicating retrorectus repair for large defects is ensuring posterior coverage. Options for this include conventional closure of the posterior sheath with adequate mobility, preservation and use of the hernia sac as a midline bridge or bridging with a biologic mesh such as BP (Figure 3) [40]. There are several issues with using the hernia sac as a bridge. The sac may become devascularised and non‐viable during dissection, small defects must be meticulously closed to avoid non‐composite mesh contact with underlying bowel, and the tissue quality and thickness is not uniform, potentially making it prone to tearing when sutured under tension. It should be noted however that use of peritoneal tissues remains an established and widely accepted technique in complex abdominal wall reconstruction. The peritoneum is vascularised, readily available and has demonstrated favourable outcomes in several series [41, 42] and so remains a viable option in appropriately selected cases. Bridging defects in the posterior sheath with BP offers a solution that can be tailored to the individual patient's needs regardless of tissue quality. In a study of either primary or recurrent ventral hernia repair in either contaminated wounds or cases where prosthetic mesh had failed, Limpert et al. demonstrated both safety and efficacy of bovine pericardium in a high‐risk patient population using both inlay and onlay techniques [43]. In this context, the potential advantage of BP lies in providing a durable biologic scaffold in situations where native tissue is inadequate or where avoidance of permanent synthetic mesh is desirable, particularly in the setting of contamination or infection risk [2, 43].

**FIGURE 3:**
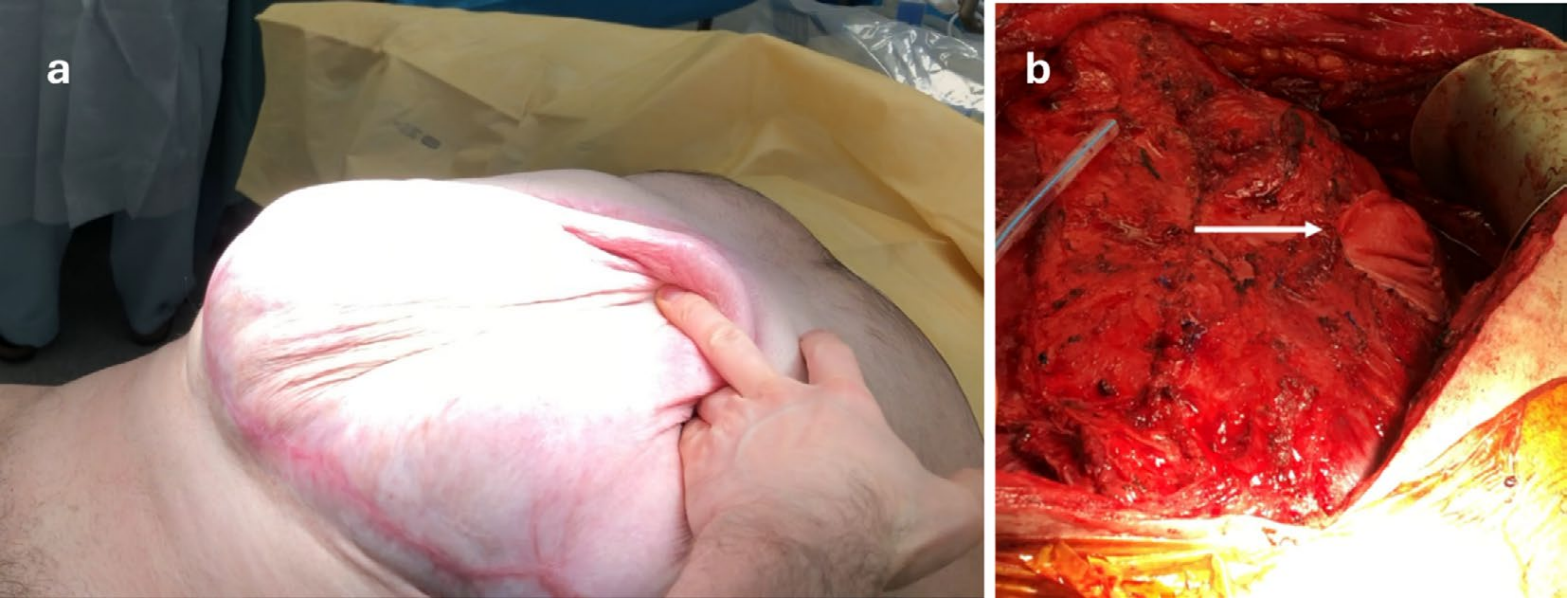
Complex ventral hernia (a) managed with transversus abdominus release (posterior component separation), repair of posterior elements with inlay bovine pericardium patch (b) facilitating retrorectus mesh placement.

### 4.4 Arteriovenous Fistula Revision

Arteriovenous fistulas (AVF) are the preferred access for haemodialysis in patients with end‐stage renal failure [44]. AVF creation has increased globally, but non‐maturation and issues with long‐term patency remain an issue [45, 46]. Conventional management for non‐maturation and stenosis includes anastomotic revision, accessory vein ligation, and fistuloplasty respectively, with mixed outcomes [47]. BP patches are used in AVF revision and angioplasty when autologous veins or synthetic materials are unsuitable. Current available evidence demonstrates high primary patency rates (up to 91% at 6 months), with maintained secondary patency up to 24 months, low infection rates, and aneurysm formation [48]. Moreover, BP has the ability to allow for early needling and has demonstrated resistance to restenosis. Options for its use include longitudinal patch angioplasty or as a tubularised graft as an alternative to PTFE (Figure 4).

**FIGURE 4:**
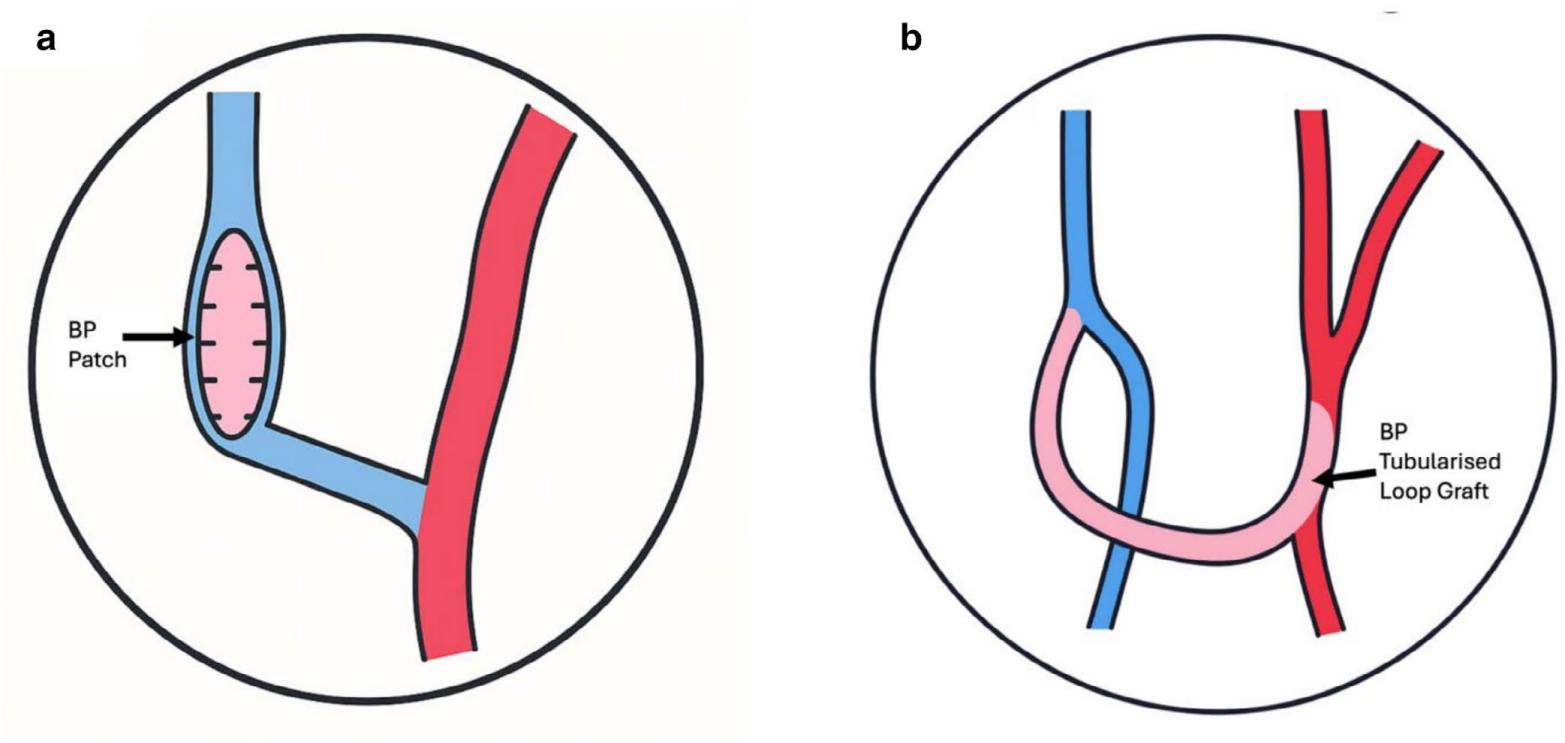
(a) Schematic diagram demonstrating longitudinal patch fistuloplasty versus (b) tubularised loop arteriovenous fistula graft.

### 4.5 Gastrointestinal Anastomotic Reinforcement

Anastomotic leak is one of the most troublesome complications in general, upper and lower gastrointestinal surgery. It is defined as a defect in the wall of the intestine at the anastomotic site leading to intra‐ and extra‐luminal compartment communication [49]. Multiple patient and technical factors including comorbidities, indication for surgery, blood supply, tension, and surgeon expertise may influence rates of anastomotic leak. A proposed method of reducing incidence of leaks is reinforcing anastomoses with an external coating material. Options including omentoplasty, fibrin sealants, collagen patches and bovine pericardium have been analysed, though predominantly in animal studies [50, 51, 52, 53, 54, 55, 56].

In their experimental pig model, Testini et al. demonstrated reduced anastomotic leak rates in both ileo‐ileal and colo‐colic interrupted, hand‐sewn anastomoses, reduced adhesion rates and no shrinkage or displacement of the BP patch [52]. Interestingly, in one group a single suture of the anastomosis was deliberately incompletely performed and the anastomosis wrapped with BP, with no leaks, stenosis, abscess or peritonitis demonstrated [52]. Moreover, biochemical studies demonstrated improved mitochondrial function and preservation of contractility and mucosal function in patched anastomoses, suggesting potentially enhanced healing with BP [52]. In canine models, improved bursting strength has also been demonstrated for anastomoses buttressed with BP patches [55, 56].

In humans, BP patches have been used to reinforce staple lines in patients undergoing Roux‐en‐Y gastric bypass and sleeve gastrectomy with mixed results [55, 57, 58]. The novel and successful use of BP for the repair of persistent cervical oesophagogastric anastomotic leaks in 7 patients following oesophagectomy for squamous cell carcinoma has also been described, with no evidence of recurrent anastomotic leakage or stenosis [53].

While these experimental findings are encouraging, clinical data in humans remain limited. Further prospective studies are required to determine whether BP reinforcement meaningfully reduces anastomotic leak rates in routine surgical practice.

## 5 Potential Novel Uses of Bovine Pericardium

### 5.1 Management of the Empty Pelvis Following Pelvic Exenteration

Pelvic exenteration is a radical, potentially curative treatment for advanced primary and recurrent pelvic malignancies [59]. Following evolution in multidisciplinary team meetings, imaging evaluation and planning as well as surgical technique, rate of R0 resection and indeed survival has improved dramatically. However, a substantial proportion of postoperative morbidity can be attributed to pelvic sepsis, ileus and perineal wound complications secondary to the void or empty pelvis that follows pelvic exenteration [60]. Numerous methods have been proposed to fill or exclude the pelvic dead space, including breast prostheses, myocutaneous flaps, omentoplasty, obstetric balloons, silicone tissue expanders, lipofilling, vascularised colonic flaps and biologic meshes [61, 62, 63, 64].

While it has been used in vascular reconstruction, currently there are no reports of BP being used to reconstruct the pelvic floor, fill the empty pelvis or bridge bony defects following pelvic exenteration [65]. The mechanical strength, resistance to infection, and capacity for biological integration suggest that BP may represent a useful material for reconstructing the pelvic floor or excluding small bowel from the pelvic cavity following exenteration [66]. Its pliability allows it to conform to irregular pelvic defects while its biological properties may reduce the risk of chronic infection compared with synthetic materials [67]. However, clinical data in this setting are currently lacking.

## 6 Conclusions

Bovine pericardium represents a versatile biologic material with expanding applications across general surgery. Its favourable mechanical properties, handling characteristics, and ability to perform in complex or potentially contaminated environments make it an attractive option for reconstruction in vascular, diaphragmatic, and abdominal wall surgery.

However, current evidence is largely derived from observational studies and small case series and high‐quality comparative data remain limited. While early outcomes are promising, further prospective studies are required to better define long‐term durability, optimal preparation methods and its role relative to other biologic and synthetic materials.

Greater understanding of these factors will help guide appropriate patient selection and optimise outcomes in complex surgical reconstruction.

## Author Contributions

**Michael J. Solomon:** writing – review and editing, supervision. **Kilian G. M. Brown:** writing – review and editing. **William A. Ziaziaris:** conceptualization, writing – original draft, writing – review and editing, visualization. **Jerome M. Laurence:** writing – review and editing, supervision, conceptualization. **Makaela Bowman:** writing – review and editing, visualization. **Paul D. Morris:** writing – review and editing.

## Funding

The authors have nothing to report.

## Conflicts of Interest

The authors declare no conflicts of interest.

## Data Availability

Data sharing not applicable to this article as no datasets were generated or analysed during the current study.
